# Compact, cost-effective and field-portable microscope prototype based on MISHELF microscopy

**DOI:** 10.1038/srep43291

**Published:** 2017-02-24

**Authors:** Martín Sanz, José Ángel Picazo-Bueno, Luis Granero, Javier García, Vicente Micó

**Affiliations:** 1 Universidad de Valencia, Departamento de Óptica y Optometría y Ciencias de la Visión, Burjassot, C/Doctor Moliner 50, 46100, Spain

## Abstract

We report on a reduced cost, portable and compact prototype design of lensless holographic microscope with an illumination/detection scheme based on wavelength multiplexing, working with single hologram acquisition and using a fast convergence algorithm for image processing. All together, MISHELF (initials coming from Multi-Illumination Single-Holographic-Exposure Lensless Fresnel) microscopy allows the recording of three Fresnel domain diffraction patterns in a single camera snap-shot incoming from illuminating the sample with three coherent lights at once. Previous implementations have proposed an illumination/detection procedure based on a tuned (illumination wavelengths centered at the maximum sensitivity of the camera detection channels) configuration but here we report on a detuned (non-centered ones) scheme resulting in prototype miniaturization and cost reduction. Thus, MISHELF microscopy in combination with a novel and fast iterative algorithm allows high-resolution (μm range) phase-retrieved (twin image elimination) quantitative phase imaging of dynamic events (video rate recording speed). The performance of this microscope prototype is validated through experiments using both amplitude (USAF resolution test) and complex (live swine sperm cells and flowing microbeads) samples. The proposed method becomes in an alternative instrument improving some capabilities of existing lensless microscopes.

Microscopy is one of the most used imaging modalities in scientific research being, in particular, light microscopy the favorite one in biomedicine due to its inherently non-invasive nature. However, the usage of microscopes in the field-setting is limited due to several factors such as cost, size and easy-to-use meaning that the development of new medical tools/devices aimed to provide early and accurate diagnosis is nowadays considerably required. For instance, global healthcare and point-of-care diagnosis are demanding reduced-cost and portable medical devices in addition to easy-to-use by a non-trained person, high reliability and rapid response for diagnosing^1^. Under this perspective, cost-effective, light-weight, compact and portable imaging devices greatly improve rapid and accurate diagnosis in the field-setting.

New developments in different optoelectronic fields such as micro-optics, solid state lasers, electronic detection devices and optical fibers, just to cite a few, have enabled cost-effective, portable and miniaturized versatile devices for biomedical applications^2^,^3^,^4^,^5^,^6^,^7^,^8^,^9^,^10^,^11^,^12^,^13^,^14^,^15^,^16^,^17^,^18^,^19^,^20^, some of them working in a lensfree scheme^2^,^3^,^4^,^5^,^6^,^7^,^8^ while others operating as smartphones implementations^9^,^10^,^11^,^12^,^13^,^14^,^15^ or using conventional but miniaturized microscopes architectures^16^,^17^,^18^,^19^,^20^. It is worth to note that all of them are complementary devices allowing different capabilities depending on the application they are aimed to. For instance, lensfree devices are ideal for field applications since they offer small-size and light-weight digital imaging portable platforms; miniaturized microscopes provide high resolution and magnification values with great flexibility coming from setting different objective lenses which perfectly match with disease diagnosis and clinical studies; and smartphone-based microscopes enable exceptional communication capabilities and affordability for telemedicine and remote diagnostics in resource-limited settings.

Lensfree bright field microscopy (or lensless holographic microscopy - LHM) derives from a digital implementation of the Gabor’s invention of holography reported in the middle of the past century^21^,^22^. Essentially, LHM proposes an extremely simple layout where a point source of coherent light illuminates the sample and the diffracted wavefront is recorded by a digital sensor^23^. Typically, the point source is implemented using a spatial aperture (or pinhole) in combination with a laser beam^5^,^23^,^24^ but additional illumination procedures such as LED^25^ and SLED^26^ sources, pulsed laser radiation^27^ and illumination through GRIN lenses^28^ or fiber optics^29^ were also successfully reported.

There are two opposite layouts with particular significance in LHM^30^. In the first one (Gabor’s like implementation), the sample is placed in close proximity with the illumination point source while the digital sensor becomes farther^23^,^24^,^25^,^27^,^28^. Thus, the sample’s diffraction pattern is magnified (geometric projection) at the digital sensor plane with typical magnification factors ranging from 5X to 20X depending of the layout distances thus relaxing the sampling requirements of the digital sensor. This experimental arrangement provides, after numerical reconstruction, similar images to the ones reported in conventional holographic microscopy regarding magnification, field of view (FOV) and resolution when considering objectives up to medium numerical aperture (NA) values (∼0.4–0.5 NA)^31^, although superior quality images concerning resolution were also attained^32^,^33^,^34^. The second implementation places the sample in close contact with the digital recording device and the illumination point source remaining further from them^2^,^3^,^4^,^6^,^7^,^8^,^29^. In this case, no geometric magnification factor is introduced (∼1X range) so the resolution limit is usually restricted by the geometrical constraints of the detector and not by the diffraction limit. However, the allowable FOV becomes in the whole sensitive area of the detector. Nonetheless and although superresolution approaches can be implemented to decrease effective pixel size^29^,^35^,^36^,^37^, it provides a modest resolution limit incoming from relatively low NA values (∼0.2 NA range) because of the geometrical constraints imposed by the pixel characteristics. Although these two opposite experimental arrangements are frequently implemented, intermediate cases have also been reported in the literature^5^.

Regarding the drawbacks, both LHM as well as Gabor’s original idea of holography suffer from several issues such as coherent noise, weak diffraction assumption and twin image problem^38^. In that sense, coherence noise can be reduced by using partially coherent sources^25^,^26^,^39^ or by overlapping several in-line holographic recordings^40^,^41^. Weak diffraction assumption (the most restrictive issue in Gabor holography since it can prevent image formation) restricts the range of samples which can be imaged by LHM but it can be circumvented by both reinserting an external reference beam (like in a Leith&Upatnieks configuration) or by applying special implementations of phase-shifting holography^38^,^42^. And finally, twin image problem raises as a direct consequence of the in-line configuration and it can be avoided in LHM by using two different strategies: phase-shifting^43^,^44^ and phase retrieval^45^,^46^,^47^,^48^,^49^,^50^ algorithms. Phase shifting is based on the relative phase variation between both interferometric beams^38^,^42^,^43^,^44^ while phase retrieval can be conducted from iterative^45^,^46^,^47^ as well as from deterministic^48^,^49^,^50^ algorithms.

Twin image problem is usually addressed using several sequential recordings in time by, for instance, multi-height recording using glass cover slips of different thicknesses^29^ or by mechanical displacement of the camera^45^ or tuning the illumination wavelength^46^ or defocusing the image^47^. Time dependence for phase retrieval was avoided by Waller *et al*.^50^ by proposing a clever method based on the transport of intensity equation (TIE) where the axial defocus between images is provided by the intrinsic chromatic aberration of imaging lenses. Thus, using broadband illumination and a single RGB color camera snap-shot, three axially slightly defocused images (one in focus, one under and other over focus) corresponding with the three color camera channels are recorded at once and enter as inputs in the TIE algorithm. Their method is ultimately limited by the camera acquisition frame rate when imaging fast dynamic biological events, thus avoiding the sequential recording of multiple misfocused images needed in previous methods.

The concept introduced by Waller *et al*.^50^ has been recently adapted to quantitative phase imaging in LHM^51^,^52^. While the first is based on a refractive principle (axial chromatic aberration by refraction), the latter falls on diffractive constraints (wavelength dependence of diffraction). Thus, by illuminating the sample with three RGB color-coded simultaneous beams, three diffraction Fresnel patterns are recorded at once in a single color camera snap-shot. After demosaicing the multiplexed in-line hologram to separate the three wavelength-coded channels, digital image processing based on numerical propagation and phase retrieval algorithm finally yield in a phase contrast image of the inspected sample. In addition, a novel and fast convergence algorithm was introduced improving the capabilities of the technique (named by us as Multi-Illumination Single-Holographic-Exposure Lensless Fresnel–MISHELF microscopy) concerning quantitative phase imaging, resolution and processing time^52^.

In this manuscript, we expand up MISHELF microscopy concept by building a compact, cost-effective and field-portable microscope based on a generalized MISHELF concept. On previous implementations^51^,^52^, RGB proposed scheme is based on the fact that the illumination is tuned with the maximum spectral sensitivity of the RGB camera channels. Thus, the color-coded RGB images are directly read from the RGB camera channels since the crosstalks can be disregarded. However, compact and cost-effective RGB coherent illumination sources are not available in the market thus disabling this MISHELF scheme as useful in the field-setting where low-cost solutions are demanded. Instead of this, inexpensive multiplexed coherent illumination is available from the laser diode source included, for instance, in some Blu-ray optical units. Within this compact and small diode can, three solid state lasers are included for reading compact discs (infrared–IR–light), digital versatile discs (red–R–illumination) and Blue-ray discs (blue–B–wavelength). Because of these three wavelengths are not tuned with the RGB maximum sensitivities of regular color cameras, each wavelength contributes at the three camera channels and direct demosaicing cannot be applied to extract the multiplexed Fresnel patterns as in the RGB implementation. Moreover, different magnified and shifted Fresnel patterns are recorded since the IRRB emitters are axially and transversally slightly shifted; so additional software capabilities must be incorporated. We have built a compact microscope prototype using this inexpensive triple laser source in addition with a modified MISHELF algorithm to accommodate this version of MISHELF microscopy with detuned illumination/detection scheme and scaled/shifted Fresnel patterns. All together allows MISHELF microscopy to be generalized and stepped forward, to be implemented in a field-setting portable platform using reduced cost components (not as bench-top system within a well-controlled environment), and to be of significant importance in developing countries and environments with limited resources.

## Results

### Prototype manufacturing

**Figure 1:**
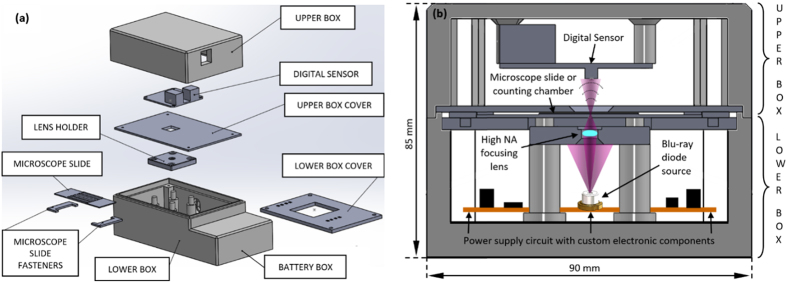
Prototype design. (**a**) explosion view of the main components and (**b**) assembly diagram of a central cross-section of the prototype with external dimensions.

**Figure 2:**
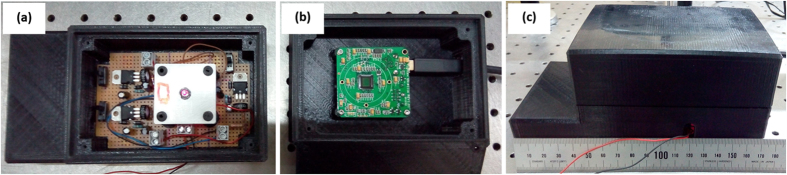
Manufactured prototype: pictures at the lab of (**a**) the lower box, (**b**) the upper box and (**c**) a global view of the prototype.

To demonstrate the capabilities of our imaging platform, a microscope prototype has been designed (using commercially available CAD software platform) and built (using Fused Deposition Material with ABS for most of the components and mechanization process for the rest). An exploded drawing of the different components as well as a global assembly scheme are included at Fig. 1 while the manufactured prototype is presented at Fig. 2.

Essentially, the prototype consists in two independent parts (upper and lower boxes), one for holding the camera (upper box) and the other (lower one) for the rest of the components of the layout. The idea is to have easy access to enter the sample without the use of movable trays inserted through lateral slots; thus, both boxes can be separated, the sample placed on the location aimed to, and the boxes joined again for sample analysis. The upper box has a lateral hole for extracting the USB camera cable towards the computer and a plastic cover to protect the board level camera (the cover is not visible in the pictures). This cover contains a clear optical window in front of the camera sensitive area to allow light passing. The camera can be regulated a few millimeter in height for properly accommodating its position to the geometrical parameters of the system (essentially magnification and resolution). The upper box is placed face to face with the lower one and the upper box cover remains closely but not in contact with the microscope slide. The lower box contains a customized board circuit acting as power supply of the three laser diodes.

The emission power of the three sources has been previously equalized according to the spectral sensitivity of the camera for providing comparable image intensity at the three camera channels. Potentiometers, voltage regulators, on/off switches and other electronic components integrate the customized board circuit. The high NA focusing lens is assembled onto a lens holder plate and supplemented away in height from the laser diode can using four pillars. The distance between the lens and the laser can is around 22 mm to allow beam spreading and homogenization over the whole lens diameter. The lens focuses the three laser beams into a set of three high NA slightly laterally and axially shifted point sources just below the sample. The lens can be moved up and down a few millimeters to both properly set the position of the point sources regarding the sample (see the “d_λ_-z” distance of the figure included at subsection “Field-portable MISHELF microscope design: experimental layout” of the Methods) and to define a reasonable magnification factor when also considering the distance between the camera and the sample (“z” distance of the same figure). As example of a real configuration in the prototype, (d-z) ≅ 0.5 mm and z = 7 mm meaning that M ≅ 15X. Note that this value is slightly different for each illumination source due to the slightly different axial position of each point source after lens focusing.

Finally, a cover plastic plate closes the lower box (not visible in the pictures). This cover plate has an internal hole having the shape of the lens holder plate just to allow close proximity between the sample and the lens. And the sample is placed on a microscope slide which is hold to the cover plate using two fasteners with adjustable positions. In addition, the lower box contains a small lateral hole for inserting the external power supply (9 volts) and a supplementary space for the case of using power batteries.

### Calibration of the prototype

**Figure 3:**
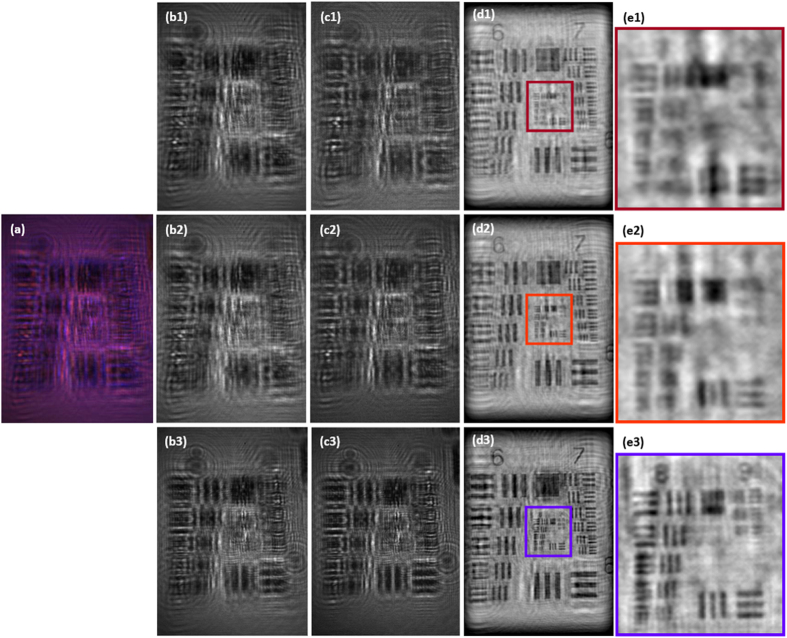
Results for MISHELF microscopy using detuned illumination/detection with the USAF test: calibration results of the prototype. The 1st row (**a**) includes the wavelength multiplexed in-line hologram while the 2nd one (**b**) depicts the three RGB color channels provided by the camera. The 3rd row (**c**) includes the retrieved holograms for each true illumination wavelength without image equalization. And the 4^th^ (**d**) and 5^th^ (**e**) rows present the in focus images of the test and a magnified area of the inner high resolution part, respectively.

**Figure 4:**
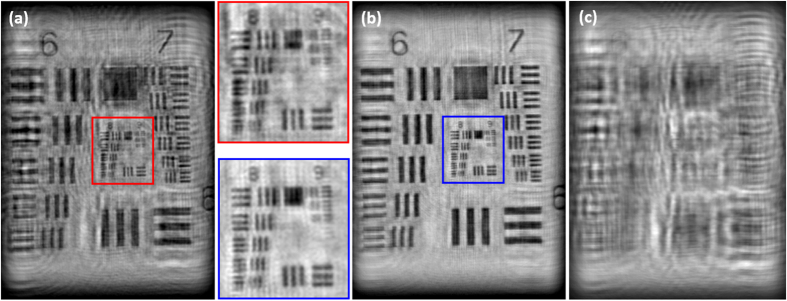
Results for MISHELF microscopy using detuned illumination/detection with the USAF test: final results comparison. The full frame images (and their magnified inner parts) for (**a**) B-LHM and (**b**) our IRRB scheme, and (**c**) the intensity distribution obtained at the twin image plane for the B-LHM case showing no presence of the twin image contribution.

Our prototype is now calibrated using a static USAF resolution test target. The USAF test case is useful to explain some significant stages of the MISHELF algorithm presented in the Methods section. The calibration results (intensity images) are included at Figs 3 and 4. The recorded hologram using the three simultaneous illuminations (IRRB sources) is included in Fig. 3(a). Then, the RGB camera channels are obtained by demosaicing that hologram. The resulting images are included at the second column of Fig. 3 (images b1-b2-b3). But because of the detuned illumination/detection scheme, each wavelength contributes to all recording channels; so the information becomes mixed and needs to be separately recovered. The third row presents the retrieved holograms for each true illumination wavelength (images c1-c2-c3). Note as the B channel (b3) is quite similar to the true B illumination hologram (c3) since the crosstalks at the B camera channel incoming from IR and R illuminations are very weak. But this is not the case of the R and G channels where the contributions of IR and R wavelengths are quiet similar in intensity at those channels. In addition, the third row also brings into light the slightly different scales (magnifications) and positions (shifts) of the in-line holograms for the true IRRB illuminations. In particular, we have computed a magnification factors of M = 14.2X/15.3X/16.1X for IR/R/B, respectively, values that are in good agreement with the previously established theoretical one (M ≅ 15X).

The retrieved true illumination holograms (images c1-c2-c3) are equalized considering both magnification/shift compensation and center-to-border illumination homogenization. Then, each hologram is separately brought into focus and the resulting full frame images are included in the fourth row (images d1-d2-d3) of Fig. 3 while the fifth row (images e1-e2-e3) depicts a magnification of the high resolution (central) area of the fourth row images. One can see as the resolution is improved from G8-E3 (G and E incoming from Group and Element, respectively) of the USAF resolution test target until G9-E2 corresponding with 3.1 μm (323 lp/mm) and 1.7 μm (575 lp/mm) for the IR and B illuminations, respectively, and for the largest camera direction. These values define a NA value of around 0.24 according to the theoretical definition (R ∼λ/NA) and for the shortest illumination wavelength.

Finally, these three images enter into the algorithm concerning single fused image synthesis and phase retrieval. The final result is included at Fig. 4 in comparison with the result obtained when using single B illumination wavelength. One can see as the resolution limit is maintained at both images (around 1.7 μm under the previously stated conditions) but the IRRB image gains in background homogeneity and contrast as consequence of the noise averaging and twin image elimination. According to the USAF retrieved area, the FOV included at Fig. 4(a) and (b) is around 225 × 300 μm, approximately. To clearly state that twin image is eliminated, Fig. 4(c) presents the intensity distribution obtained when the recovered IRRB complex amplitude is numerically propagated until the twin image plane for the B illumination case. One can see as MISHELF microscopy eliminates the twin image: the intensity distribution contains no USAF in-focus image and only the misfocused pattern obtained by numerical propagation to that plane. On the other hand, conventional B-LHM produces a twin image contribution (not included in Fig. 4) which is equal but inverted and conjugated to the one presented in Fig. 4(a).

### Experiments with dynamic samples

The prototype has also been tested via two different types of dynamic samples. The first validation involves an alive swine sperm sample. The sperm cells have approximately a head’s width of 6 × 9 μm, a total length of 55 μm, and a tail’s width of 2 μm on the head side and below 1 μm on the end. The biosample is introduced by micropipetting into a commercially available counting chamber (100 μm thickness). The sperm cells move along the chamber and the inspection area is around 300 × 300 μm. Due to the fast movement of the sperm cells, we have replaced the previous camera (Mightex SMN-C050-U) by a faster one (AVT Mako G-419, 90fps at 1024 × 1024 pixels, 5 × 5 pixel pitch). Thus, the recording time for a single frame is around 10 ms and only limited by the frame rate of the camera.

**Figure 5:**
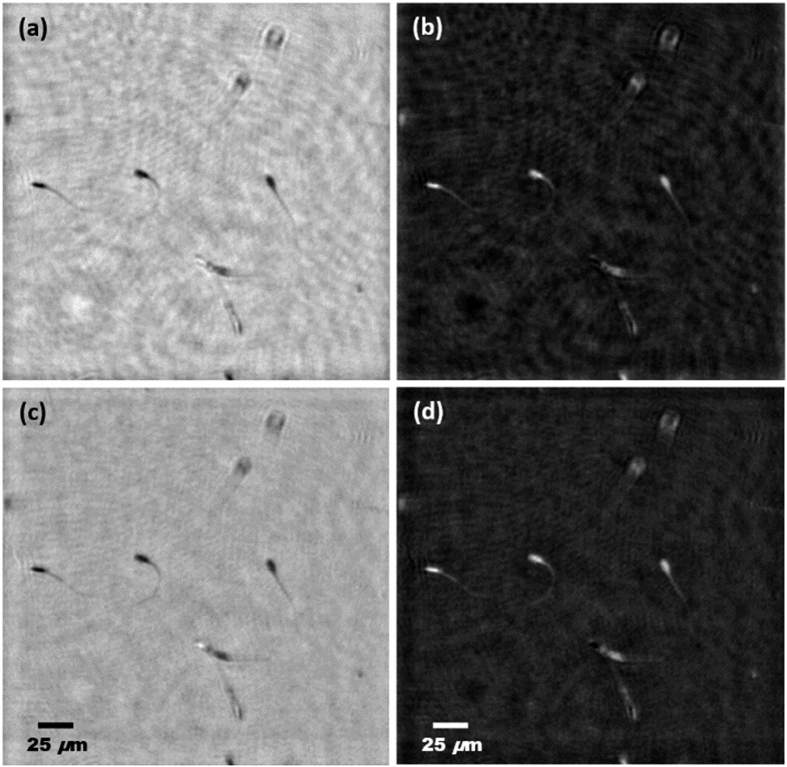
Results for MISHELF microscopy using detuned illumination/detection with alive sperm biosample. Images (**a,b**) and (**c,d**) represent, respectively, the phase distribution when considering B-LHM and our IRRB scheme for positive [(**a**) and (**c**)] and negative [(**b**) and (**d**)] phase contrast visualization. In addition, the whole image sequence for each case is included from Video1.mov to Video4.mov, respectively (2.4MB per video movie).

Figure 5 shows the experimental results concerning the phase distribution of the sperm biosample. Images (a)-(b) are for the single B wavelength illumination case while (c)-(d) are the ones obtained with the proposed MISHELF microscopy method for positive [(a) and (c)] and negative [(b) and (d)] phase contrast visualization modes. In addition, the images at Fig. 5 are the first frames of a recording sequence of images which are separately included in video movies from Video1.mov to Video4.mov files. The total recording time was 2 seconds and the video movies are played at 12.5 fps to slowly see in detail the sperm cell’s movement. From Fig. 5, one can see as the proposed approach improves the phase stability from a qualitative point of view since the background noise of the phase images is reduced as consequence of the averaging provided by the MISHELF image multiplexing. It can also be seen as the contrast (cell’s visibility against background) of the cells becomes enhanced and the tails of the cells are clearly visible for all of them while they are barely resolved in most of the cells with single B illumination LHM. Finally, the obtained resolution is in concordance with the calibration experiment (∼1.7 μm) since, as one can see in the images, the tails of the sperm cells are visible at the head’s side (∼2 μm) while they are not resolved on the other side (<1 μm).

**Figure 6:**
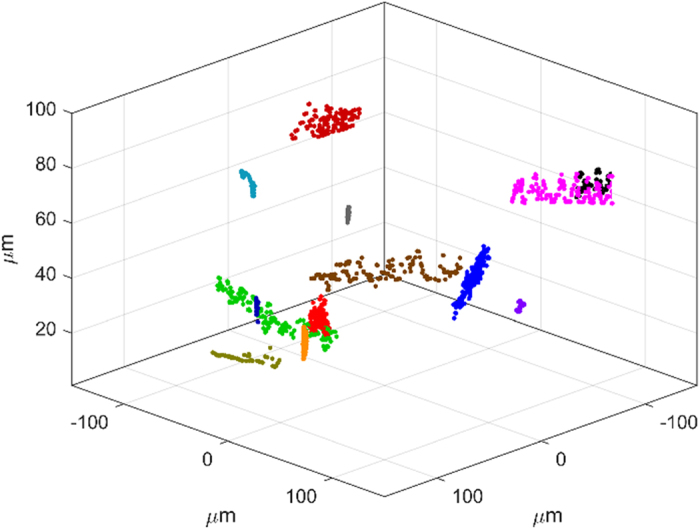
Results for MISHELF microscopy using detuned illumination/detection with alive sperm biosample: 3D plot of the spermatozoid trajectories. The generation of the trajectories is included in Video5 file (Video5.mov, 6.9 MB).

As it can be seen from the previous videos, there are several sperm cells that are defocused because they are moving at different planes inside the counting chamber. All those spermatozoids can also be refocused by numerical propagation, thus selecting the axial plane where they are in focus. This information is very useful to retrieve a 3D trajectory of each sperm cell in the 3D volume defined by the thick counting chamber^53^,^54^,^55^,^56^,^57^,^58^,^59^. The result is included in Video5.mov where a set of 13 trajectories are evaluated, one per each spermatozoid moving along the FOV. Figure 6 depicts the last frame of the 3D plot containing all the extracted trajectories.

**Figure 7:**
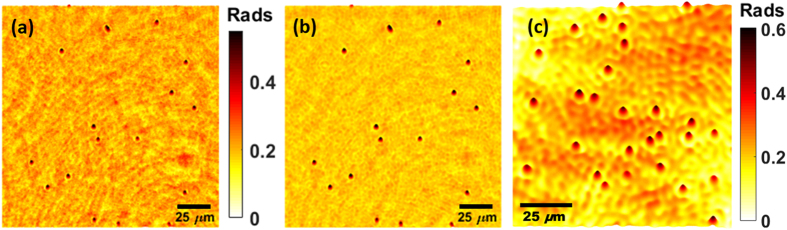
Results for MISHELF microscopy using detuned illumination/detection with microbeads: 3D plots of the phase distributions for (**a**) B-LHM, (**b**) our IRRB scheme and (**c**) a Match-Zehnder interferometric layout assembled at the lab. (**a**) and (**b**) images share the same scale bar (the one place in the middle) in rads. The whole image sequence for the lensless experiments is included in Video6 movie file (Video6.mov, 2.3 MB).

Our second experiment including a dynamic sample considers standard monodisperse polystyrene microspheres in aqueous suspension (Polybead^®^ Microspheres) with a diameter of 3.00 ± 0.07 μm according to the theoretical specifications of the manufacturer. For this experiment, we have used the same sensor as in the USAF experiment (Mightex USB3.0 color camera). Figure 7 includes a comparison of the experimental results in 3D-plot visualization mode of the retrieved phase distributions for: (a) the single B illumination case and (b) our IRRB scheme. In order to validate these results, Fig. 7(c) includes the 3D view of the phase values retrieved by a regular Match-Zehnder interferometric configuration assembled at an optical bench in the lab. Although they are not the same flowing microbeads included at (a) and (b), it is an image coming from the same sample in origin which is prepared analogously to the lensless experiment one; so a similar phase profile should be obtained. The interferometric layout uses a He–Ne laser to illuminate the microbeads which are imaged by a microscope lens (0.15NA Olympus UMPlanFl) onto a CCD camera (Basler A312f, 582 × 782 pixels, 8.3 μm pixel size, 12 bits/pixel). When combined with a tilted reference beam, an off-axis hologram is recorded and the complex amplitude information of the microbeads can be retrieved by using conventional and widely-known operations (Fourier transform of the hologram, spatial filtering at the Fourier plane, inverse Fourier transform, aberration phase compensation and 3D plots). Note that it is a single frame image in contrast with the video recording provided for the MISHELF microscopy experiment and included in Video6.mov file. We have used a 0.15NA objective since it is the nearest lens at the lab having a similar NA value to the estimated one provided by MISHELF microscopy prototype for the shortest sensor direction and, thus, the retrieved phase values should be roughly similar. This estimation is derived from Fig. 4b where the last resolved element in the horizontal direction is around G9-E1 defining a resolution limit of ∼2 μm and meaning a NA value of around 0.17 when considering the shortest sensor direction (1920 pixels).

To validate the results from a quantitative point of view, we have computed the averaged background values of all the images as well as the maximum phase step introduced by the microbeads. Then, their difference will be the phase disturbance caused by the microspheres. The results are summarized in Table 1 where the phase step introduced by the microbeads shows a high concordance between the phase values provided by the proposed IRRB method and the quantitative phase information retrieved from conventional digital holographic microscopy. For completeness, the last row at Table 1 also includes the Standard Deviation (STD) values of the background. These values are also useful to show the improvement in phase stability provided by MISHELF microscopy since the background averages and fluctuates at around lower levels than for the single B illumination case.

## Discussion and Conclusions

MISHELF microscopy is a powerful and versatile tool with a strong potential in biomedical applications. Although previous implementation^52^ reported on a similar background concept, it was only a proof-of-concept and preliminary validation at the lab of a specific implementation of the technique which is very far to become a commercially available and tangible reality. This manuscript provides a step forward on MISHELF microscopy since: i) it reports on the general frame of the method regarding hardware (illumination/detection scheme) and software (complete algorithm description), ii) it is not a technique running at the lab since a real portable prototype has been manufactured and characterized, and iii) it presents experimental results with not only static samples but dynamic experiments with alive samples. Those capabilities are based on relevant changes in hardware components (illumination source and focusing lens) and software (algorithm adaptation to the new detuned scheme) with respect to ref. 52, changes allowing miniaturization of the technique/device to make it a fact in the field-setting.

Thus, a new design and prototype of LHM based on wavelength multiplexing, working with single hologram acquisition and using a fast convergence algorithm for image processing is presented along this manuscript. All together, the reported MISHELF microscopy enables single-shot improved quantitative phase imaging by twin-image elimination in LHM. This is a direct consequence of the simultaneous multiple illumination and a robust and powerful phase retrieval algorithm incoming from a single camera snap-shot as input. Our field-portable, compact and cost-effective MISHELF microscope achieves micrometric range lateral resolution over an inspection area which is comparable with the FOV provided by microscope objectives having similar NA and resolution values. However, this value can be accommodated for higher NA values until reaching the maximum theoretical NA value defined by the illumination source (0.6 NA in this prototype but a reasonable real one of ∼0.4 NA). Additional advantages come from the compactness and weight (∼300 grams) of the prototype. Due to its compact design and the possibility to operate without external power supply, our MISHELF microscope can be particularly interesting and useful for point-of care diagnosis or in the field-setting.

The resolution limit in LHM depends on the selected configuration (sample in close contact with the point source or with the recording device). MISHELF microscopy belongs to the Gabor’s like implementation techniques meaning that no geometrical constraints of the detector (sampling theorem) affect the resolution limit because of the geometrical magnification factor introduced by the layout. Thus, the resolution limit is defined from its classical definition of R ∼λ/NA meaning that the best resolution is achieved when considering the shorter (B illumination) wavelength (see images e1-e2-e3 at Fig. 3) and the higher NA coming from the largest sensor direction (in case it will be rectangular). Our MISHELF microscope prototype keeps the best achievable resolution incoming (B illumination) at the final image (see Fig. 4) while uses the other two illuminations for twin image elimination and quantitative phase improvement regarding contrast, noise and halos. This is accomplished by applying a fast convergence algorithm based on illuminating the sample at once with three simultaneous coherent lights (RGB at^52^ and IRRB in the proposed realization) and recording a single multiplexed hologram with a color camera at video rate speed. This wavelength multiplexing introduces a limitation at the proposed method: it is needed that the intensity distribution of the sample will contain the same spatial profile for all the illumination wavelengths to be used. In other words, the color object information is sacrificed to achieve twin image elimination and improved quantitative phase imaging. So an inherent drawback of the proposed MISHELF microscopy technique is the use of color selective samples such as, for instance, red blood cells or samples with different color-coded attached particles (fluorophores, dyes, etc.). On those cases, wavelength-dependent differences in absorption will result in different hologram intensities for each illumination wavelength or, even worst, the absence of spatial information at those highly absorptive areas. To mitigate this effect, the intensity at those penalized wavelengths can be increased to finally get a weak image from which, although it will not be useful for defining the best final resolution limit, it can be used as part of the phase retrieval algorithm to provide a good quality reconstruction. In another direction, it is possible to think in a multi-wavelength illumination source containing more than three lights (see for instance SpectraTec X from Blue Sky Research) so the most appropriated three ones could be lighted on for the illumination thus minimizing the previous problem.

The proposed prototype supposes a proof-of-principle technology in the field setting which is oriented towards sperm analysis applications (cell motility, concentration evaluation, trajectory analysis, etc.). Aimed to this, we designed the prototype to be as much compact as possible and adapted to this specific application. As a result, a small size and robust prototype which can be the background of an improved miniaturized microscope’s family in next future has been validated. Obviously, the advantages for our application field can be converted into penalizations for different ones. Although some parts of the prototype can be redesigned to accommodate specific constraints for different applications, there is a strong limitation imposed by the small distance between the sample and the sensor (an inherent limitation of the used LHM layout). This fact prevents the broad use of the system since it is not possible, for instance, to use cell culture Petri dishes for investigations on living cells. Nevertheless, the proposed prototype is fully valid and useful for its general use with regular microscope slides (with or without coverslips) and regular plates/counting chambers/microfluidic platforms/etc. of similar thickness dimensions. This fact widely extends the applicability of the proposed device to its general use with this type of slides, especially in the field setting and guided by a non-trained user.

In the validation, we have conducted experiments with both static (non-moving) and dynamic (moving) samples. Static objects (USAF resolution test) allow us to calibrate the prototype in terms of layout considerations as well as in resolution, FOV and algorithmic. Dynamic samples (as fast as the acquisition frame rate of the camera) emphasize the power and versatility of the proposed method for a wide variety of applications such as sperm monitoring and sorting^53^,^54^,^55^,^56^ and particle tracking^57^,^58^,^59^, just to city the same examples where experiments are included in this manuscript. Future applications can be directed, for instance, to merge MISHELF microscopy with microfluidic platforms (holographic optofluidic lensless microscopy ref. 37) thus allowing flow cytometry, waterbone parasite analysis, etc., and also for telemedicine applications in resource limited environments.

## Methods

This section introduces MISHELF microscopy by detuning the illumination and the detection, thus expanding up our previous implementation^52^ to a general framework where a given illumination wavelength contributes to more than a single recording channel. Due to this, MISHELF microscopy is optimized and generalized and a field-portable device is built in virtue of this generalization. Optimized in the sense of miniaturization, thus improving its compactness and robustness, and by using low cost elements while providing high quality images. And generalized because the overall framework of MISHELF microscopy is addressed by considering a detuned scenario between illumination and detection stages. Moreover, the selected configuration causes a slightly different magnification and lateral shift in the recorded holograms and this fact must be digitally compensated to get a high quality final image. Thus, the previously reported fast convergence algorithm is now modified to include all those changes, resulting in a more robust, powerful and complete imaging algorithm.

### Field-portable MISHELF microscope design: experimental layout

**Figure 8:**
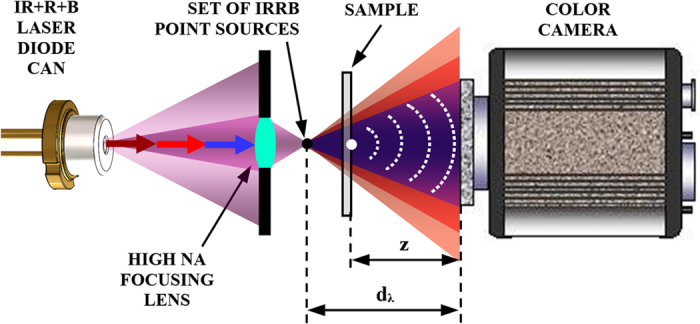
Layout for MISHELF microscopy with detuned illumination/detection using IRRB/RGB multiplexing.

The experimental layout of the generalized MISHELF microscopy concept is depicted at Fig. 8. A coherent illumination source emitting in Infrared (IR)-Red (R)-Blue (B) laser lights (780–660–405 nm, respectively) and a conventional RGB digital recording device define the detuned illumination/detection scheme. This IRRB laser source is removed from a Blu-ray optical unit (Sony model KES-400AAA PS3) and consists on three individual diode lasers which are slightly separated both axially and transversally (typically by a tenths of millimeter). In order to homogenize the illumination (irregular beam shape emitted by the diodes) as well as to equalize (different divergence of the laser diodes) and increase its NA value, we use a focusing commercial-grade plastic lens of 0.6NA. This lens forms a set of three point sources which are slightly separated according to the initial separation of the sources in the laser can and the amount of chromatic (axial and transversal) aberration of the plastic lens. This fact defines a slightly different axial and transversal position for each illumination source which also causes a wavelength-dependent magnification factor in the system (M = d_λ_/(d_λ_–z), being d_λ_ the wavelength-dependent distance between each point source and the digital sensor) and also a lateral shift of the recorded holograms. These two issues must be compensated in the calibration stage to finally get a high resolution quality image.

### Algorithm adaptation to field-portable MISHELF microscopy

**Figure 9:**
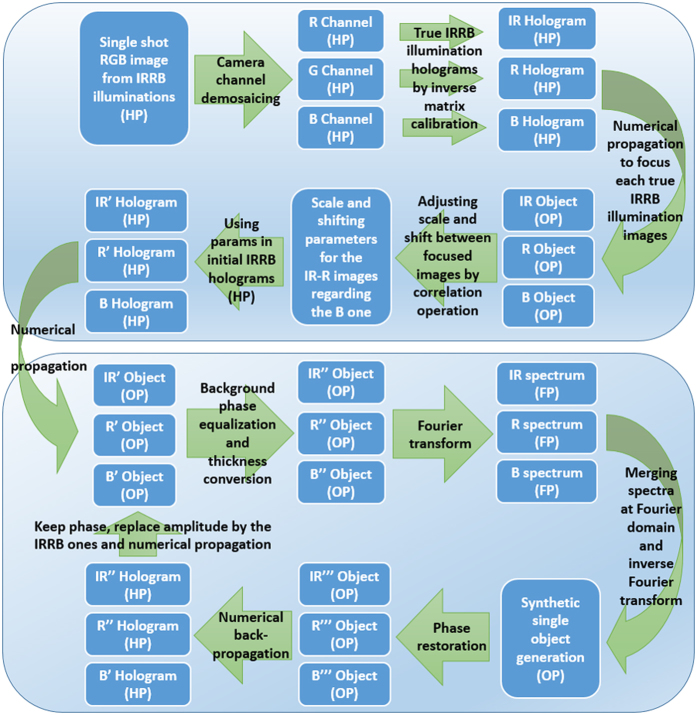
Block diagram of the MISHELF microscopy algorithm for detuned illumination/detection scheme. HP, OP and FP mean Hologram Plane, Object Plane and Fourier Plane, respectively.

Under these conditions (Fig. 8), the color camera (Mightex USB3.0 SMN-C050-U, CMOS sensor type, 2560 × 1920 pixels, 2.2 × 2.2 μm pixel pitch) for the USAF resolution test case records the three in-line holograms but with crosstalks contributions incoming from the spectral sensitivity of the RGB camera channels at the used IRRB illumination wavelengths. A global workflow of the MISHELF microscopy algorithm adapted for this detuned illumination/detection scheme is presented in Fig. 9. Essentially, the algorithm is divided into two main blocks (upper and lower parts at Fig. 9). The first one relates with a pre-digital preparation of the holograms before entering into the second block which performs phase retrieval by applying a fast convergence algorithm. Although it is not our aim to describe in detail the algorithm, we will comment its main points in the following paragraphs.

The first block starts with the recording of the RGB in-line hologram in a single camera snap-shot: the sample becomes illuminated with the trichromatic IRRB source and one multiplexed RGB hologram is recorded by the color camera. This RGB hologram is then separated into its three elementary RGB channels where, in general, each one of the illuminations will contribute to each camera channel with different efficiency depending on the specific spectral sensitivity for that wavelength at this channel. And these cross-talks must be removed before continuing; otherwise, the quality of the reconstructed images will be restricted. To easy understand it, let us assume that crosstalks are not removed. Then, each hologram will contain information from the two holographic images (real and virtual) incoming from the useful wavelength at such channel in addition with the two holographic images incoming from the other two illuminations. On one hand, this fact reduces the dynamic range of the hologram; so contrast is penalized. And on the other hand, there is a high background fluctuation incoming from the misfocused images when focusing at the image of interest, yielding in a high disturbance at the phase distribution.

To avoid final quality image restriction, crosstalks must be removed. After demosaicing to separately extract the RGB information, the real IRRB holograms corresponding with the true illumination wavelengths are computed. This process must be applied to each in-line recorded hologram and it is performed by using device calibration, that is, defining a matrix whose coefficients are the amount of IRRB light going to each RGB channel. After that, the true IRRB holograms are brought into focus by numerical propagation allowing three in focus images of the object but with different magnifications and shifts as we have previously noticed. By using correlation operation between those in focus images, all the images are perfectly equalized in magnification and centering. The scale and shift computed factors are then applied to the true IRRB in-line holograms to remove the different magnifications and displacements caused by the three slight differently placed point sources. As result, three true IRRB in-line holograms (IR’R’B holograms at Fig. 9) are obtained as they were provided by a single point source (like the pinhole used in ref. 52) with three different illumination wavelengths. In addition, illumination profile homogenization is applied to each hologram to compensate the lower intensity at the border of the images. This image equalization stage needs to be done once and it involves basic (subtraction) digital image processing which can be easily implemented before sample insertion.

After that, these three IR’R’B in-line holograms enter in the second block regarding phase iteration algorithm. The process is quiet similar to the one described in ref. 52 but a few words are included here for paper comprehensiveness. The phase iteration algorithm starts with the definition of the initial amplitudes incoming from each IR’R’B hologram as the square root of their intensity. These three IR’R’B amplitudes are propagated to its best focus (generating IR’R’B’ object images at Fig. 9) and the resulting propagation distances are stored in the computer’s memory. Those propagation distances are slightly different one to each other due to, on one hand, the thickness of the sample’s slide slightly modifies the effective axial position of the sample because of the wavelength dependence in refractive index and, on the other hand, the wavelength dependence on the spatial phase evolution in the Fresnel regime. Regarding the different numerical methods for computing the digital reconstruction of the diffraction integral, we have selected to solve the diffraction Rayleigh-Sommerfeld integral using convolution operation since it allows an effective and economical calculation without any approximation.

These three in-focus IR’R’B’ images of the object are then used to synthesize a single fused image through the following steps. First, the global background phase of the three in-focus images is forced to be equal just to avoid phase cancellation effects when mixing their spectra at the Fourier domain. Also, phase distribution is converted into thickness profile (ΔL = Δφ λ/2π, being ΔL the Optical Path Length and Δφ the phase distribution) to avoid wavelength dependence in the phase distribution. This process generates the IR“R“B” in-focus images of Fig. 9. Second, we perform digital fast Fourier transform (FFT) of each IR“R“B” in-focus image (IRRB spectrums at Fig. 9). Third, the three spectra are then used to synthesize a new single spectrum incoming from a weighted combination of them. By weighted we mean that, since the information of the IR spectrum is fully included at the R one and both are also included at the B spectrum, the addition of the three spectra must be weighted by a mask in order to avoid low spatial frequency enhancement. And fourth, inverse FFT of the generated mixed spectrum recovers an improved image of the sample (synthetic single object generation step at Fig. 9), improved in the sense of noise averaging due to the mixing of the three images into a single one.

This synthetic single object is used to generate a new set of in-focus images (IR’“R’“B”‘ objects at Fig. 9) coming from a phase restoration from the single object thickness distribution by applying the inverse previously applied transformation (Δφ = 2π ΔL/λ). These three new in-focus images are then numerically back propagated until the hologram plane (IR“R“B’ holograms at Fig. 9) taken into account the three slightly different propagation distances (one per each wavelength) previously stored at the computer’s memory. And from these IR“R“B’ holograms, a new set of IRRB in-line holograms are generated by retaining the phase distributions while replacing the amplitude ones by the square root of the initially recorded hologram intensities (output of the first block: IR’R’B holograms). The previous cycle starts (propagation to focus the object, thickness conversion, FFT, merging the spectra and inverse FFT, etc.) again and, after a few number of iterations (typically 2 iterations), a final image containing complex amplitude information of the sample is obtained with improved capabilities since it contains no information about the twin image and exhibits better image quality concerning noise, halos and contrast. Moreover, the single spectrum generation process allows fast convergence in the phase retrieval algorithm since information of the three channels is mixed together in each iteration.

### Swine sample preparation

The swine sample was received and ten micro liters of the swine’s sample were taken and added into the oval tube which is placed at the ISASPC heated plate (a plate from PROISER R+D S.L. that keeps the optimum temperature for tubes and slides at constant temperature). The oval tube were kept for 10 min at 37 °C. After that, the semen sample were diluted with Acromax using ⅓ dilution rate (Acromax^®^ is a diluent from Gestión Veterinaria Porcina S.L.). Three micro liters of the dilution were used to load the counting chamber having 100 microns thickness. And the stuffed counting chamber is placed into the prototype for MISHELF microscopy characterization.

## Additional Information

**How to cite this article:** Sanz, M. *et al*. Compact, cost-effective and field-portable microscope prototype based on MISHELF microscopy. *Sci. Rep.* **7**, 43291; doi: 10.1038/srep43291 (2017).

**Publisher's note:** Springer Nature remains neutral with regard to jurisdictional claims in published maps and institutional affiliations.

## Supplementary Material

Supplementary Legens of the Videos

Supplementary Video 1

Supplementary Video 2

Supplementary Video 3

Supplementary Video 4

Supplementary Video 5

Supplementary Video 6

## Figures and Tables

**Table 1:** Phase values comparison for B-LHM, IRRB MISHELF microscopy and conventional Match-Zehnder interferometric configuration when using a microbeads sample.

	B-LHM	IRRB MISHELF	Interferometric layout
Background value (rads)	0.18	0.18	0.20
Maximum phase value (rads)	0.59	0.59	0.60
Microbeads phase step (rads)	0.41	0.41	0.40
Background STD (rads)	0.033	0.017	0.054
