# Supplementary material for: Compact, cost-effective and field-portable microscope prototype based on MISHELF microscopy
Source: Sci Rep. 2017 Feb 24;7:43291. doi: 10.1038/srep43291 (PMC5324169; doi:10.1038/srep43291)
Supplement: Supplementary Legens of the Videos [file srep43291-s1.doc]

**Title of the manuscript:**

Compact, cost-effective and field-portable microscope prototype based on MISHELF microscopy

# Manuscript ID:

# SREP-16-39689A

# Authors list:

# Martín Sanz, José Ángel Picazo-Bueno, Luis Granero, Javier García and Vicente Micó

# Legends for video files:

# Video1.mov: Positive phase contrast distribution for B-LHM using the swine sperm sample

# Video2.mov: Negative phase contrast distribution for B-LHM using the swine sperm sample

# Video3.mov: Positive phase contrast distribution for MISHELF microscopy using the swine sperm sample

# Video4.mov: Negative phase contrast distribution for MISHELF microscopy using the swine sperm sample

# Video5.mov: Retrieved 3D plot of the spermatozoid trajectories from MISHELF microscopy

# Video6.mov: Comparison between the 3D views of the phase distributions from B-LHM and MISHELF microscopy
